# Abnormal functional asymmetry and its behavioural correlates in adults with ADHD: A TMS-EEG study

**DOI:** 10.1371/journal.pone.0285086

**Published:** 2023-05-25

**Authors:** Amir Avnit, Samuel Zibman, Uri Alyagon, Abraham Zangen

**Affiliations:** 1 Department of Life Sciences, Ben-Gurion University of the Negev, Beer-Sheva, Israel; 2 Zlotowski Centre for Neuroscience, Ben-Gurion University of the Negev, Beer-Sheva, Israel; University of La Laguna, SPAIN

## Abstract

**Objectives:**

Abnormal functional brain asymmetry and deficient response inhibition are two core symptoms of attention deficit hyperactivity disorder (ADHD). We investigated whether these symptoms are inter-related and whether they are underlined by altered frontal excitability and by compromised interhemispheric connectivity.

**Methods:**

We studied these issues in 52 ADHD and 43 non-clinical adults by comparing: (1) stop-signal reaction time (SSRT); (2) frontal asymmetry of the N200 event-related potential component, which is evoked during response inhibition and is lateralised to the right hemisphere; (3) TMS-evoked potential (TEP) in the right frontal hemisphere, which is indicative of local cortical excitability; and (4) frontal right-to-left interhemispheric TMS signal propagation (ISP), which is reversely indicative of interhemispheric connectivity.

**Results:**

Compared to controls, the ADHD group demonstrated elongated SSRT, reduced N200 right-frontal-asymmetry, weaker TEP, and stronger ISP. Moreover, in the ADHD group, N200 right-frontal-asymmetry correlated with SSRT, with TEP, and with symptoms severity. Conversely, no relationship was observed between ISP and N200 right-frontal-asymmetry, and both TEP and ISP were found to be unrelated to SSRT.

**Conclusions:**

Our results indicate that abnormal frontal asymmetry is related to a key cognitive symptom in ADHD and suggest that it is underlined by reduced right-frontal excitability.

## 1. Introduction

Attention deficit hyperactivity disorder (ADHD) is a common neuropsychiatric disorder characterised by excessive inattention, hyperactivity, and impulsivity; either alone or in combination. ADHD, affecting ~7.2% of children worldwide [1], had previously been thought to be limited to youth and thus the vast majority of the literature is based on studies with children. However, ADHD persists into adulthood in 40 to 60% of cases [2–4], which results in a worldwide prevalence of ~3.4% in individuals over 18 years of age [5].

While ADHD has been associated with a multitude of cognitive, neurobiological, and genetic factors, its precise neuropsychological underpinnings are currently unclear [6–11]. A large body of evidence indicates a deficit in response inhibition in ADHD, which has been proposed to constitute a core determinant of this disorder [12–14]. Response inhibition is defined as the ability to deliberately withhold a pre-potent, routine, or dominant response [15, 16], and is critical for controlling impulsive, inappropriate, or irrelevant responses [17].

Commonly, response inhibition is studied using the stop-signal task (SST) [18, 19], in which participants perform accelerated responses to a “Go” stimulus in a simple discrimination task. In some of the trials, a “Stop” signal is presented following the “Go” stimulus, requiring participants to inhibit their ongoing response. The SST provides an estimate of response inhibition—the stop-signal reaction time (SSRT)—which is the time required for successful inhibition of the motor response to the “Go” stimulus [18, 19]. Relevantly, SSRT has been found to be prolonged in ADHD compared to non-clinical individuals, indicating poorer response inhibition [20].

Response inhibition is a lateralised function that requires the specialisation of the right frontal cortex [15, 21–32]. Accordingly, response inhibition (either successful or unsuccessful) is reflected by the N200 event-related potential (ERP) component: a negative deflection occurring around 200 milliseconds following the eliciting stimulus (e.g., a “Stop” signal) and distributed over the right frontal hemisphere [33, 34]. Importantly, N200 amplitude is positively associated with response inhibition performance in both ADHD and non-clinical populations, and is reduced in ADHD individuals, signifying less efficient response inhibition processing [35–39].

One influential account of ADHD is the right-hemisphere account, which implicates a right-hemispheric deficit in this disorder, and states that its symptoms arise from the disruption of lateralised right-hemispheric processes related to response inhibition, as well as to the regulation of attention and arousal [40–42]. However, in recent years, accumulating data has indicated abnormal functional asymmetry in ADHD, suggesting that the observed right-hemispheric deficit in this condition may be better explained by the ratio of activation between the two hemispheres, which may serve as an endophenotype of this disorder—that is, the asymmetry account of ADHD. Evidence supporting this account are derived from studies comparing the asymmetry of spontaneous Alpha-band oscillations in ADHD and control individuals, measured during resting-state, which indicate an abnormally reversed pattern of left-asymmetry (i.e., increased activity in the left compared to the right hemisphere) in ADHD [43–46].

While previous asymmetry studies of ADHD have mainly focused on spontaneous Alpha-band oscillations, the role of asymmetry abnormalities in ADHD-related cognitive and clinical symptoms is yet to be understood; most prominently with regard to a deficit central to its pathology as response inhibition, reflected by the right-lateralised N200 SST component. To investigate this, we hypothesised, drawing from the asymmetry account, a direct link between aberrant frontal N200 asymmetry and a response inhibition deficit in ADHD. Consequently, we expected to find reduced N200 right-asymmetry among ADHD than in non-clinical individuals, which is positively correlated with response inhibition performance, measured as SSRT. Similarly, as asymmetry abnormalities have been indicated as central to ADHD neuropathology, we expected to observe a relationship between N200 asymmetry and ADHD clinical symptoms severity.

While suggested as central to ADHD neuropathology, the underlying mechanism of asymmetry abnormalities in ADHD is currently not-well-understood. Relevantly, functional asymmetry has been indicated to arise when an area in a given hemisphere is strongly activated, leading to the transmission of interhemispheric inhibitory signalling to the homologous region of the contralateral hemisphere through the corpus callosum (CC), the main neural pathway connecting the two cerebral hemispheres [47–51]. Hence, we hypothesised that aberrant functional asymmetry in ADHD may stem, at least in part, from diminished excitability of the dominant hemisphere (i.e., the right hemisphere in the case of response inhibition) and/or by compromised functional interhemispheric connectivity. This notion of aberrant interhemispheric communication in ADHD is supported by findings of atypical functional interhemispheric connectivity in this disorder (measured as interhemispheric spectral coherence), most prominently in frontal regions [52–57]; however, its relationship with asymmetry abnormalities in this disorder is currently unclear.

In order to measure cortical excitability and functional interhemispheric connectivity, we used the combination of EEG with transcranial magnetic stimulation (TMS), a non-invasive neuromodulation technique able to depolarise neurons and trigger action potentials [58–60]. First, the magnitude of the TMS-evoked potential (TEP) in a stimulation target region following administration of single-pulses is considered to reflect local cortical excitability [58, 61–64]. Second, TEP is transmitted contralaterally, which can be used to measure the interhemispheric signal propagation (ISP). ISP is the ratio between cortical activation in the stimulated area and its homologous region in the contralateral hemisphere, and is considered to reversely reflect interhemispheric connectivity [47, 59, 65, 66].

Here, we measured EEG responses to single TMS pulses delivered to the right-frontal cortex of both ADHD and non-clinical individuals. We expected to find reduced TEP in the ADHD than in the control group, indicative of diminished right-frontal excitability. We also expected to observe increased ISP in the ADHD group, indicative of reduced frontal interhemispheric connectivity. Lastly, in order to investigate the underlying role of these possible neuromarkers of ADHD in asymmetry abnormalities, we examined their relationships to the asymmetry of the N200 SST component. To the best of our knowledge, this is the first attempt to examine a relationship between ERPs measured during task performance, and TEPs, measured in a non-task-related context. Furthermore, by measuring ISP, this study is one of the first to use TMS-EEG for examining functional interhemispheric connectivity in ADHD, and the first to do so outside of the motor cortex.

To conclude, while previous studies have indicated abnormal functional asymmetry in ADHD, its relationship with response inhibition deficiency, a core cognitive symptom of this condition, as well as with ADHD clinical symptomology, is yet to be understood. To investigate this, we combined EEG measures with the SST to examine whether the asymmetry account can describe deficient response inhibition in adult ADHD, as well as the severity of clinical symptoms in this condition. Furthermore, to study the currently not-well-understood neuronal basis of asymmetry abnormalities in ADHD, we combined TMS with EEG to investigate frontal cortical excitability and interhemispheric connectivity abnormalities in adult ADHD, and evaluate their relationship with compromised frontal asymmetry in this disorder.

## 2. Materials and methods

### 2.1 Participants

Fifty-two ADHD and 43 control participants were recruited to this study by distribution of ads throughout the Ben-Gurion University of the Negev campus and via mass university e-mail. Participants were a part of a larger, clinical study; trial registration: Trial to Evaluate the Efficacy of the HLPFC Coil Deep Transcranial Magnetic Stimulation System in Treating Attention Deficit and Hyperactivity Disorder (ADHD) in Adults, https://clinicaltrials.gov/ct2/show/NCT01737476, ClinicalTrials.gov identifier NCT01737476. A few of the findings presented here, which are not related to this study’s main subject (i.e., asymmetry abnormalities in ADHD), were previously published elsewhere [67, 68]. Participants had no history of neurological disease or psychiatric disorders (other than ADHD), and no history of taking anti-psychotic, anti-depressive, or mood stabilizing drugs, nor of drug or alcohol abuse. Potential participants received information concerning the study requirements over the telephone, and the existence of a previous diagnosis of ADHD was verified before proceeding. Next, they were screened by a senior psychiatrist using a semi-structured interview (SCID) to verify ADHD diagnosis according to DSM-V criteria and to rule out any other axis I or personality disorders. No minimum score of the Conners’ Adult ADHD Rating Scale (CAARS) or any other additional metric was required for participation. Participants were instructed to refrain from using psychoactive medication (e.g., methylphenidate) starting one week prior to the study and throughout participation. The study protocol was approved by the Soroka Medical Centre Helsinki Committee and all participants signed an informed consent form prior to participation.

Four ADHD and one control participant were excluded from the study due to either poor SST EEG data quality (i.e., >50% trials rejected due to artefacts) or poor performance of the SST (i.e., <35% successful inhibition trials or not being able to finish the task). Overall, 48 ADHD (38 males) participants, aged 21–46 years (mean = 26.54; SD = 3.75), and 42 non-clinical control participants (23 males), aged 22–32 years (mean = 25.90; SD = 2.23) were included in this study. For the TMS-EEG analysis, three ADHD and four control participants were additionally excluded due to poor data quality.

### 2.2 Clinical assessment

Severity of ADHD symptoms was assessed using the Conners’ Adult ADHD Rating Scales Self-report Long-version (CAARS-S:L) [69], and ADHD symptoms severity was defined as the total symptoms scale standardised score (t score), according to the CAARS norms.

### 2.3 Procedure

All participants filled a demographic questionnaire and the CAARS-S:L. Next, EEG was measured during: (1) 3 minutes of resting-state (eyes closed), followed by (2) performance of a computerised SST task (~20 min), and finally (3) administration of single-pulse TMS (~10 min).

### 2.4 Stop-signal task

The computerised visual SST was conducted similar to as previously described [18, 19]. In brief, in 480 experimental trials, participants were required to respond to a”Go” stimulus in a simple discrimination task, and in one quarter of the trials (i.e., 120 out of a total of 480 experimental trials) a “Stop” signal followed the “Go” signal, requiring participants to inhibit their motor response.

**Fig 1:**
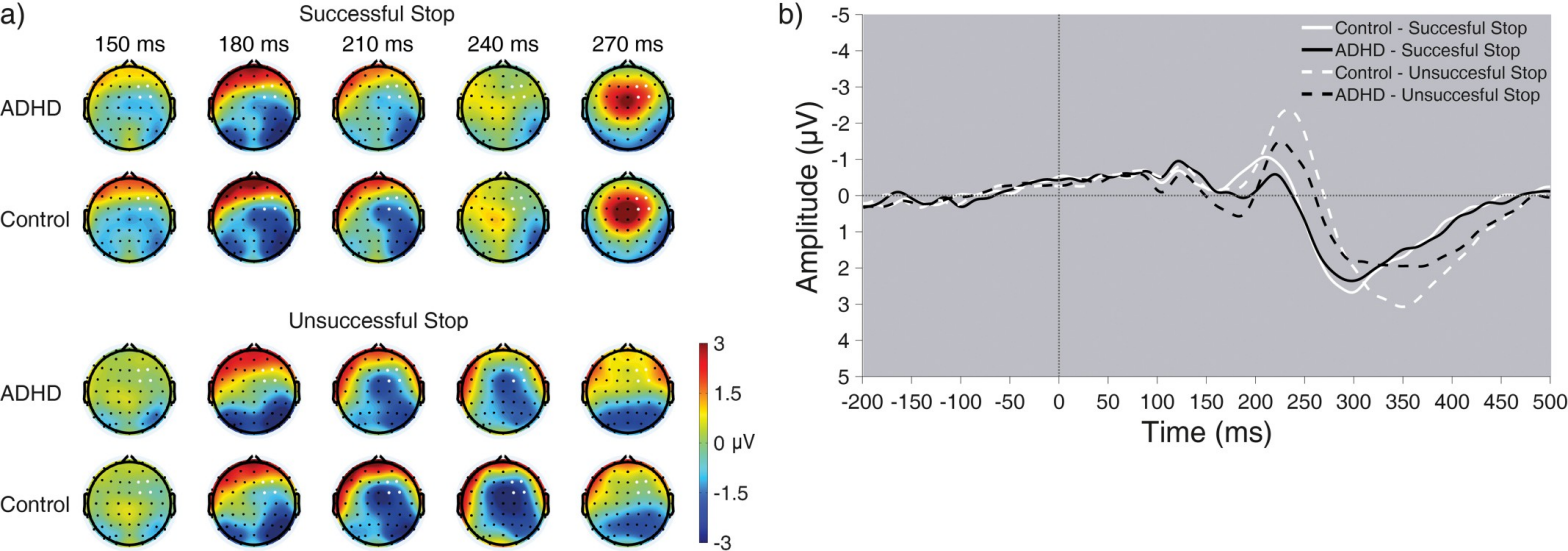
SST N200. a) Grand-averaged topographic distribution of ERPs, time-locked to the “Stop” signal, at time points of interest. Electrodes comprising the Task-ROI are marked in white. b) Time course of grand-averaged ERPs, time-locked to the “Stop” signal, in the right side of the Task-ROI.

In each trial (Fig 1), a visual stimulus (an "X" or an "O"; i.e., “Go” stimulus) was presented for 1,000 milliseconds (ms) or until response. Participants were required to respond as quickly and accurately as possible to the “Go” stimulus by hitting one of two corresponding keyboard buttons using either their left or right index finger. Response buttons were counter-balanced across participants. In 25% of trials, a “Stop” signal (a white square placed over the “Go” stimulus) was presented after a variable stop-signal delay (SSD) following the “Go” stimulus, requiring participants to inhibit their ongoing response (i.e., “Stop” trials). The shorter the SSD, the easier it is to successfully inhibit the motor response to the “Go” stimulus; the longer the SSD, the harder it is to inhibit the response. Except for practice trials, in which SSD was held constant at 300 ms, SSD was initially set to 500 ms and adjusted by a staircase tracking procedure: After each successful stopping the SSD was extended by 50 ms (making the next “Stop” trial more difficult); after each unsuccessful stopping the SSD was shortened by 50 ms (making the next “Stop” trial easier).

The task began with 12 practice trials, for which the first six trials did not include a “Stop” signal (i.e., “Go” trials), while 75% and 25% of the last six trials were “Go” and “Stop” trials, respectively. During practice, participants received feedback at the end of each trial, presented on the screen for 1,000 ms. Following, participants underwent 480 experimental trials. All trials were presented in random order. To eliminate anticipation, each experimental trial ended with a variable inter-trial interval of either 1,100, 1,300, 1,500, 1,700 or 1,900 ms, appearing in pseudo-random order, each occurring in one fifth of trials. In “Go” trials in which the participant failed to respond, a beep sound was presented at the end of the trial (i.e., 1,000 ms following the onset of the “Go” stimulus), reminding the participant to remain focused on the task.

Only participants which demonstrated a percentage of erroneous responses given a “Stop” signal in between 35–65% of “Stop” trials were included in the analysis, as large deviations from 50% successful inhibition are in violation with the assumptions of the independent horse-race model, on which the stop-signal task is based upon [70]. The percentage of erroneous response given a “Stop” signal was nearly 50% for both groups (ADHD group: mean = 49.20%, SD = 1.48; control group: mean = 49.21%, SD = 2.92; N.S.), indicating that the tracking procedure was successful. “Go” trials with RTs shorter than 200 ms were considered as anticipatory responses and excluded from all analyses. Response inhibition was estimated as SSRT, which was calculated using the integration method [70]: For each participant, “Go” trial RTs were ranked in ascending order, and SSRT was calculated as the nth RT minus the mean SSD, with the nth RT corresponding to the rank determined as N (number of correct “Go” trials) X P(response|stop signal); that is, the number of “Go” trials times the probability of an erroneous motor response to the “Go” signal given the presentation of a “Stop” signal.

### 2.5 TMS administration

TMS was applied using a Magstim-Rapid Stimulator (Magstim, Dyfed, UK) inducing biphasic pulses via a figure-eight coil, with a 70-mm diameter of each wing. Participant’s resting motor threshold (RMT) was determined by stimulation of the contralateral abductor pollicis brevis (APB) muscle and finding the minimal intensity that produces visible muscle responses following at least 5 out of 10 pulses, separated by 5 seconds [71]. Thereafter, in order to target the stimulation region, the intersection of the coil was positioned, using a Magstim coil holder, directly above electrodes FC4 and F4 in the right hemisphere. Finally, 50 single pulses were administered at the intensity of 120% of the RMT, with an inter-stimulus-interval of 5 seconds (ADHD group: mean = 77.2% of maximal stimulator intensity, SD = 12.0; control group: mean = 76.6%, SD = 8.8; N.S.). Participants wore earplugs as protection and in order to mask coil click sound and reduce auditory-evoked cortical activation.

### 2.6 EEG recording

EEG was recorded via a TMS-compatible 64-channel Waveguard^TM^ cap and amplifier (ANT neuro, Enschede, Netherlands), and data were acquired using ASA™ version 4.7.3 (ANT neuro, Enschede, Netherlands). The Cz electrode was used as a common reference (re-referenced offline to the average), and the PO6 electrode as ground. Impedance was kept below 10 kOhm in all electrodes. The signal was digitised at 2048 Hz using a 24-bit AD converter.

### 2.7 EEG preprocessing

All EEG data was processed offline using the EEGlab toolbox for MATLAB [72] and custom MATLAB scripts.

#### 2.7.1 Stop-signal task

First, data was down-sampled to 512 Hz. Then, a bandpass (1–100 Hz) and a notch (45–55 Hz) finite impulse response (FIR) filters were applied (EEGlab function pop_eegfiltnew); the second of which used to eliminate 50 Hz AC main power supply interference. Next, data of successful and unsuccessful “Stop” trials was segmented from −1000 to 1000 ms relative to “Stop” signal onset. Following, baseline correction was applied (200 to 0 ms before “Stop” signal onset; pop_rmbase). Then, noisy channels and segments were removed using both automatic algorithms (pop_rejspec; probing trials including 20–40 Hz activity which exceed a threshold of -100-25 dB) and manual inspection. Following, eye blinks and vertical eye movements were removed using infomax independent component analysis (ICA; pop_runica), as described in [72]. Then, a second, 1–40 Hz bandpass FIR filter was applied (pop_eegfiltnew). Next, removed channels were interpolated (eeg_interp). Thereafter, data was re-referenced to the common average. Then, additional artefact cleaning was conducted by discarding trials containing outlier values (exceeding -70-70 μV; pop_eegthresh) or improbable data (exceeding 4 SDs of individual or group electrodes activity probability; pop_jointprob).

Overall, preprocessing included the removal of noisy channels (ADHD group: mean = 1.04, SD = 0.89; control group: mean = 1.00, SD = 0.77; N.S.) and trials (ADHD group: mean = 24.79, SD = 10.93; control group: mean = 22.45, SD = 10.76; N.S.), as well as ICA components (ADHD group: mean = 2.58, SD = 0.90; control group: mean = 2.38, SD = 0.94; N.S.).

#### 2.7.2 TMS-evoked potential

First, Data of the first 40 ms post-pulse (0 to 40 ms) was discarded, because it contains a massive electrical artefact as well as dominant lateral muscle activation [73]. Next, data was filtered via a 1–45 Hz bandpass FIR filter (EEGlab function pop_eegfiltnew). Following, data was segmented from −1000 to 1000 ms relative to the TMS pulse. Then, channels and segments containing excessive TMS-related artefacts, which jeopardise the integrity of ICA decomposition, were removed using both an automatic algorithm (pop_jointprob, used for detecting electrodes exceeding 4 SDs of group or individual activity probability) and manual inspection. Following, data was re-referenced to the common average. Thereafter, an infomax ICA was conducted and non-TMS-related components (i.e., eye blinks, vertical eye movements and residual muscle artefacts) were identified and cleared semi-automatically using the TMS-EEG signal analyser plugin) [74]. Next, removed channels were interpolated (eeg_interp). Finally, baseline correction (500 to 10 ms before pulse) was applied.

Overall, preprocessing included the removal of noisy channels (ADHD group: mean = 2.69, SD = 3.04; control group: mean = 1.32, SD = 1.53; F_1,81_ = 6.37, p < .05, η_p_^2^ = .073) and segments (ADHD group: mean = 3.74, SD = 1.95; control group: mean = 3.14, SD = 1.52; N.S.), as well as ICA components (ADHD group: mean = 5.27, SD = 2.90; control group: mean = 4.21, SD = 2.49; N.S.).

### 2.8 EEG analysis

#### 2.8.1 Regions of interest and time windows

Regions of interest (ROIs) were chosen based on previous literature on the SST N200 component [75, 76] and the TMS target region. These include, in accordance with the 10–20 system: (1) a task-related ROI (Task-ROI; right electrodes: FC2, FC4, F2, & F4; left electrodes: FC1, FC3, F1, & F3), which was used for N200 analysis, and a stimulation-related ROI (Stim-ROI; right electrodes: FC4 & F4; left electrodes: FC3 & F3), which is comprised of electrodes directly under the TMS figure-eight coil intersection and was used for TEP and ISP analysis. Time windows for the Task-ROI were determined based on previous literature [75, 76] and following inspection of the N200 ERP component obtained in the current study (See Results section 3.3.1). Time windows of 190–220 and 210–260 ms post-stimulus were chosen for the successful and unsuccessful trials, respectively, as amplitude enhancement of the N200 component is typically delayed and elongated in the later (see [37, 77–79], and Results section 3.3.1). For the Stim-ROI, a time window of 50–150 ms post-pulse was used, based on previous literature [47, 80–83] and following inspection of the TEP time course obtained in the current study (see Results section 3.4.1). Importantly, an offset of 150 ms roughly matches the release duration of GABA_B_ receptors, suggested to mediate interhemispheric inhibition and brain asymmetry [84, 85].

#### 2.8.2 N200 amplitude and right-asymmetry

The N200 component was calculated as the mean voltage amplitude of electrodes in the right side of the Task-ROI. N200 asymmetry was quantified by calculating the relative amplitude at each homologous electrode pair [86], and represented as the natural-log-transformed ratio between the area under the rectified curve (AURC) of the right and left Task-ROI:

$$\mathrm{Right}‐\mathrm{asymmetry}=ln\left(\frac{{N200\;AURC}_{Right}}{{N200\;AURC}_{Left}}\right)$$

For this measure, positive scores are indicative of right-asymmetry while negative scores are indicative of left-asymmetry, and higher absolute values represent stronger asymmetry. Importantly, this method provides a degree of correction for overall power, as large individual differences may be confounded with the magnitude of the asymmetry, as well as mitigates the impact of individual differences (e.g., in skull thickness) on signal amplitude [86]. Finally, N200 amplitude and right-asymmetry were averaged separately across successful and unsuccessful “Stop” trials in the corresponding time windows, and grand-averaged for the ADHD and control groups.

#### 2.8.3 TMS-EEG

TEP was calculated as the average AURC in the right electrodes directly under the TMS figure-eight coil intersection, i.e., the right electrodes of the Stim-ROI. ISP was calculated similarly to the right-asymmetry measure, with two exceptions: (1) since TMS was delivered to the right hemisphere, the ratio of the contralateral left hemisphere and the ipsilateral right hemisphere was calculated; (2) a 10-ms delay was used for the contralateral left hemisphere to account for the time required for the induced signal to propagate from the site of the stimulation to the contralateral hemisphere [47, 87]. The following ISP calculation was used for each homologous electrode pair:

$$\mathrm{ISP}=ln\left(\frac{{TEP\;AURC}_{Left}}{TEP\;{AURC}_{Right}}\right)$$

For this measure, negative scores are indictive of greater TMS-induced activation in the ipsilateral than in the contralateral hemisphere, while higher scores signify increased signal propagation between the hemispheres. Finally, TEP and ISP were averaged separately in the corresponding time windows, and grand-averaged for the ADHD and control groups.

#### 2.9 Statistical analysis

Symptoms severity, TEP, and ISP were compared via a one-way analysis of variance (ANOVA) with Group (ADHD vs. control) as a between-subject factor. A similar analysis was used for group comparison of SST behavioural measurements: “Go” trials error rates and reaction time (RT), and “Stop” trials SSRT. N200 amplitude and right-asymmetry were analysed via a mixed-design two-way ANOVA, with Group (ADHD vs. control) as a between-subject factor and Stopping (successful vs. unsuccessful stops) as a within-subject factor.

The relationships between N200 right-asymmetry and SSRT, ADHD symptoms severity, TEP, and ISP, were assessed using multiple regression analyses for successful and unsuccessful “Stop” trials, with averaged N200 amplitude in the right and left sides of the Task-ROI used as independent measures. Thus, a model with significant R^2^ value showing relationships of reverse directionality between the activation in each side and the dependent variable (i.e., a positive β coefficient for one side and a negative coefficient for the other side) represents a significant relationship between N200 asymmetry and that dependent variable. To further investigate group differences in observed relationships between N200 asymmetry and a given dependent variable, the difference in the partial correlation between the left ROI and that dependant variable, controlling for the right ROI, in the ADHD and control groups was analysed via a Z-test between the Fisher-transformed correlation coefficients. Bivariate correlation analyses were conducted to examine the relationships between SSRT and TEP and between SSRT and ISP.

## 3. Results

Descriptive statistics of clinical and neuropsychological study variables are presented in Table 1.

**Table 1:** Means (± standard deviations) of clinical and neuropsychological study variables.

	ADHD Group	Control Group
	*n* = 48	*n* = 42
CAARS total symptoms	77.65 (10.76)	52.33 (10.42)
Stop-signal task		
“Go” error rate (%)	7.17 (4.09)	5.57 (4.02)
“Go” reaction time (ms)	594.01 (89.38)	574.93 (105.87)
P(R|S) (%)	51.21 (1.18)	51.39 (2.88)
SSRT	245.55 (42.01)	225.08 (32.01)
Successful N200 (μV)	-0.27 (1.50)	-0.96 (2.44)
Unsuccessful N200 (μV)	-1.03 (1.36)	-1.90 (2.23)

*Note*: N200 amplitudes are depicted as group grand-averages in the right side of the Task region of interest; p(R|S): percentage of “Stop” trials with erroneous responses following the presentation of a “Stop” signal.

### 3.1 Clinical assessment

As expected, CAARS total symptoms scores in the ADHD group were higher than that of controls (*F*_1,88_ = 127.70, *p* < .001, *η*_*p*_^*2*^ = .592).

### 3.2 Stop-signal task behavioural performance

“Go” error rates in the ADHD group were marginally higher than that of controls (*F*_1,88_ = 3.49, *p* = .065, *η*_*p*_^*2*^ = .038), but correct “Go” RT was not significantly different between the groups (*F*_1,88_ = .86, *p* = .356, *η*_*p*_^*2*^ = .010). As expected, SSRT of ADHD participants was significantly longer than that of controls (*F*_1,88_ = 6.61, *p* = .012, *η*_*p*_^*2*^ = .070).

### 3.3 Electrophysiology

#### 3.3.1 N200 amplitude

The spatiotemporal distribution of the N200 component is presented in Fig 1. As reported elsewhere [68], analysis of right-frontal N200 amplitude revealed main effects for Group (*F*_1,88_ = 4.44, *p* = .379, *η*_*p*_^*2*^ = .048) and Stopping (*F*_1,88_ = 29.49, *p* < .001, *η*_*p*_^*2*^ = .251), but no Group X Stopping interaction (*F*_1,88_ = .30, *p* = .583, *η*_*p*_^*2*^ = .003). That is, N200 amplitude was larger in the control (relative to the ADHD) group, and in unsuccessful (relative to successful) “Stop” trials.

#### 3.3.2 N200 asymmetry

**Fig 2:**
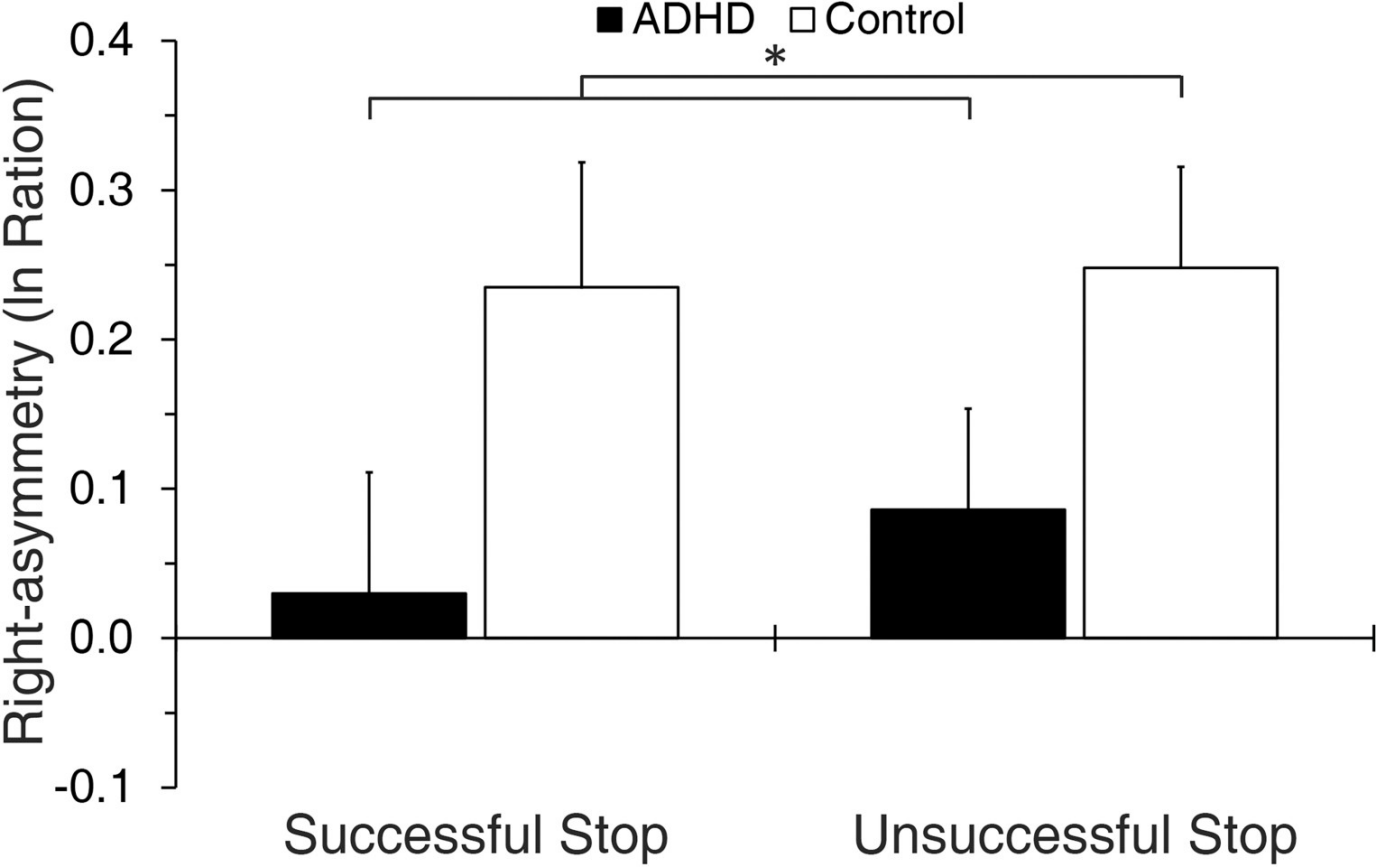
Grand-averaged N200 right-frontal-asymmetry in the Task-ROI. *p < .05. Error bars here and throughout represent one standard error from the mean.

Analysis of N200 right-frontal-asymmetry (Fig 2) revealed a main effect for Group (*F*_1,88_ = 5.06, *p* = .027, *η*_*p*_^*2*^ = .054), but no main effect for Stopping (*F*_1,88_ = .25, *p* = .618, *η*_*p*_^*2*^ = .003), nor a Group X Stopping interaction (*F*_1,88_ = .10, *p* = .757, *η*_*p*_^*2*^ = .001). That is, N200 right-frontal-asymmetry was reduced in the ADHD group across stopping success conditions.

*3*.*3*.*2*.*1 Relationship between N200 frontal-asymmetry and response inhibition*. Relationships between response inhibition performance and N200 amplitude are presented in Table 2. The analysis revealed that for successful “Stop” trials in the ADHD group, a marginally significant relationship was observed between greater right-frontal N200 asymmetry and shorter SSRT. In contrast, for successful “Stop” trials in the control group, a marginally significant relationship was observed only between increased right-sided N200 amplitude and shorter SSRT. The partial correlation between the left ROI and SSRT in successful “Stop” trials controlling for the right ROI was not significantly stronger in the ADHD group compared to the control group (*Z* = -1.21, *p* = .226). For both groups, no relationship between SSRT and N200 asymmetry in unsuccessful “Stop” trials was observed. These results indicate that while the performance of successful response inhibition is marginally related to stronger right-frontal-asymmetry in the ADHD group, and marginally related to frontal right-sided amplitude alone in the control group, the groups do not statistically differ with respect to the relationship between N200 asymmetry and response inhibition.

**Table 2:** Relationships between N200 amplitude and SSRT. Note that since N200 represents negative activation, positive β coefficients indicate that higher N200 amplitude is related to shorter SSRT, and vice-versa for negative coefficients.

Stop Success	Predictors	ADHD Group					Control Group				
		β	p	R^2^	F	p	β	p	R^2^	F	p
Successful	Right ROI	.48	.025	.11	2.78	.073	.46	.018	.13	3.04	.059
	Left ROI	-.40	.057				-.27	.159			
Unsuccessful	Right ROI	.02	.940	.06	1.32	.277	.31	.108	.07	1.36	.269
	Left ROI	.22	.365				-.16	.388			

#### 3.3.3 Relationship between N200 asymmetry and ADHD symptoms severity

Relationships between CAARS total symptoms scores and N200 amplitude are presented in Table 3. The analysis revealed that for unsuccessful “Stop” trials in the ADHD group, greater right-frontal N200 amplitude was related to increased severity of symptoms, while greater left-frontal N200 amplitude was related to reduced severity of symptoms. No significant relationships were observed in the control group. The partial correlation between the left ROI in unsuccessful “Stop” trials and CAARS total symptoms controlling for the right ROI was significantly stronger in the ADHD group compared to the control group (*Z* = -2.97, *p* = .003), supporting the conclusion that the groups differed in the extent to which the left ROI had a unique contribution to CAARS total symptoms scores, and thus that the relationship between N200 asymmetry and CAARS total symptoms scores magnitude, obtained in the ADHD group, is significantly different between the groups. These results indicate that stronger right-frontal-asymmetry during unsuccessful “Stop” trials is related to more severe symptoms in the ADHD group.

**Table 3:** Relationships between N200 amplitude and ADHD symptoms severity. Note that since N200 represents negative activation, positive β coefficients indicate that higher N200 amplitude is related to lower symptoms severity scores, and vice-versa for negative coefficients.

Stop Success	Predictors	ADHD Group					Control Group				
		β	p	R^2^	F	p	β	p	R^2^	F	p
Successful	Right ROI	-.11	.598	.03	0.64	.530	.16	.422	.03	0.59	.557
	Left ROI	.23	.290				-.21	.293			
Unsuccessful	Right ROI	-.55	.016	.20	5.73	.006	.08	.692	.04	0.77	.468
	Left ROI	.75	.002				-.23	.238			

### 3.4 TMS-EEG

#### 3.4.1 Group differences

**Fig 3:**
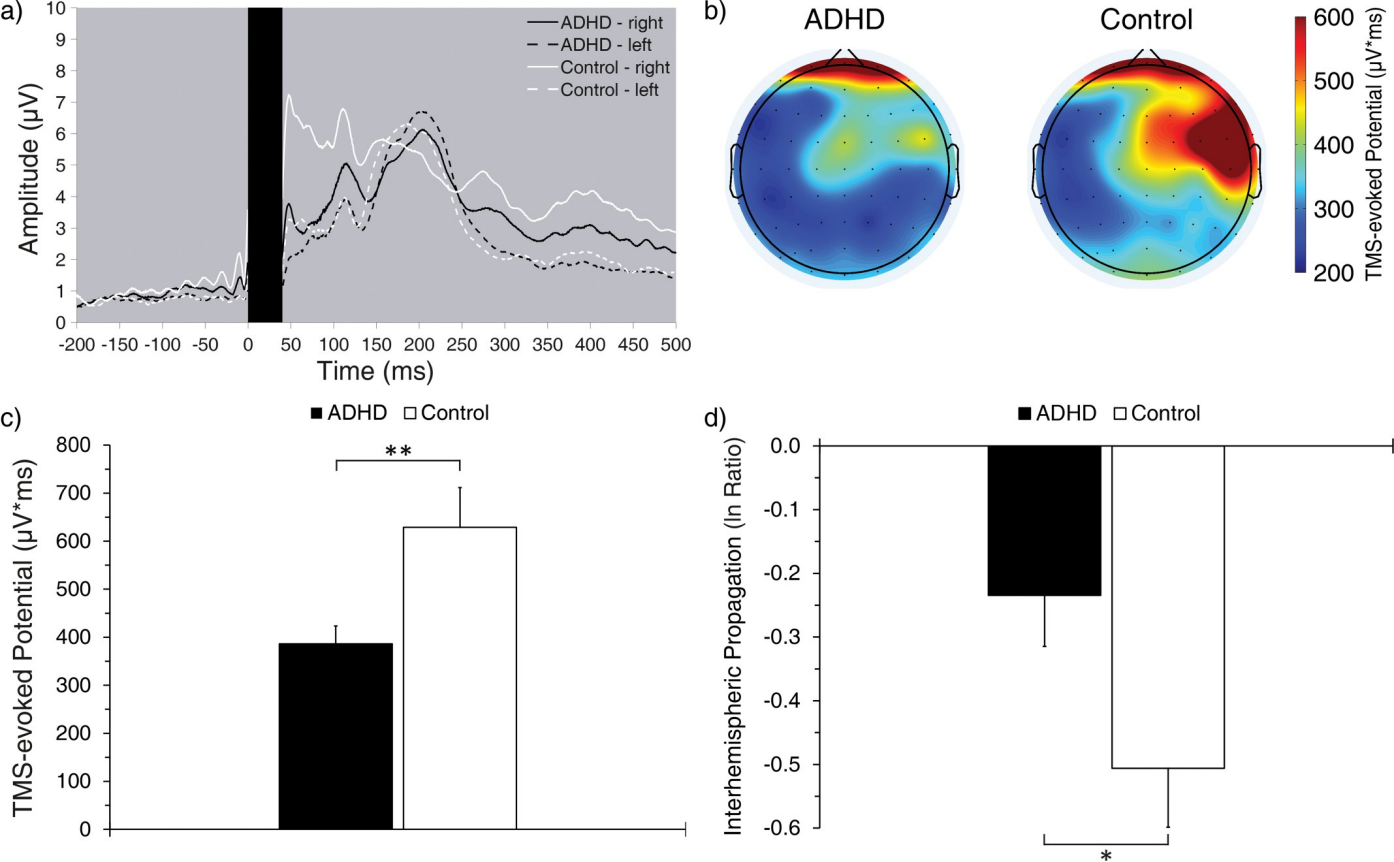
TMS-induced activation following the delivery of a single pulse to the right frontal cortex. a) Time course of grand-averaged TEP, time-locked to the TMS pulse, where the dark rectangle represents activation discarded due to containing TMS-induced artefacts (0–40 ms post-pulse). b) Grand-averaged topographic distribution of TEP. c) Grand-averaged TEP in the right side of the Stim-ROI. d) Grand-averaged ISP in the Stim-ROI. *p < .05; **p < .01.

The analysis revealed that TEP in the ADHD group was significantly weaker than that of the control group (*F*_1,81_ = 7.95, *p* = .006, *η*_*p*_^*2*^ = .089; Fig 3A–3C), while ISP was significantly stronger in the ADHD group than that of the control group (*F*_1,81_ = 4.97, *p* = .029, *η*_*p*_^*2*^ = .058; Fig 3D). These results indicate reduced excitability of the right frontal cortex and diminished frontal interhemispheric connectivity in ADHD, respectively.

#### 3.4.2 Relationships between TMS-EEG measures and N200 asymmetry

*TMS-evoked Potential*. Relationships between TEP (in the Stim-ROI) and N200 amplitude (in the Task-ROI) are presented in Table 4. The analysis revealed that for successful “Stop” trials in the ADHD group, greater right-frontal N200 amplitude was associated with stronger TEP, while greater left-frontal N200 amplitude was associated with weaker TEP. No relationships were observed in the control group, as well as in both groups for unsuccessful “Stop” trials. The partial correlation between the left ROI in successful “Stop” trials and TEP magnitude controlling for the right ROI was significantly stronger in the ADHD group compared to the control group (*Z* = 2.01, *p* = .044), supporting the conclusion that the groups differed in the extent to which the left ROI had a unique contribution to TEP, and thus that the relationship between N200 asymmetry and TEP magnitude, obtained in the ADHD group, is significantly different between the groups. These results indicate a positive relationship between N200 frontal right-asymmetry during successful stops and the excitability of the right frontal cortex in the ADHD group.

**Table 4:** Relationships between N200 amplitude and TEP. Note that since N200 represents negative activation, positive β coefficients indicate that higher N200 amplitude is related to weaker TEP, and vice-versa for negative coefficients.

Stop Success	Predictors	ADHD Group					Control Group				
		β	p	R^2^	F	p	β	p	R^2^	F	p
Successful	Right ROI	-.54	.010	.15	3.77	.031	-.05	.817	.03	0.46	.633
	Left ROI	.46	.026				-.13	.541			
Unsuccessful	Right ROI	-.16	.514	.05	1.14	.330	-.01	.951	.12	2.41	.105
	Left ROI	-.08	.752				-.34	.087			

*Interhemispheric signal propagation*. Relationships between ISP (in the Stim-ROI) and N200 amplitude (in the Task-ROI) are presented in Table 5. The analysis revealed that for unsuccessful “Stop” trials in the control group, N200 amplitude in the left side of the Stim-ROI was negatively related to ISP. No relationships were observed in the ADHD group, as well as in both groups for successful “Stop” trials. These results indicate that right-frontal-asymmetry is not related to interhemispheric connectivity in either group, and that increased frontal negative deflection in the left frontal hemisphere during unsuccessful stops in control participants is related to reduced interhemispheric propagation in the control group, and thus to increased interhemispheric connectivity.

**Table 5:** Relationships between N200 amplitude and ISP. Note that since N200 represents negative activation, positive β coefficients indicate that higher N200 amplitude is related to weaker ISP, and vice-versa for negative coefficients.

Stop Success	Predictors	ADHD Group					Control Group				
		β	p	R^2^	F	p	β	p	R^2^	F	p
Successful	Right ROI	.21	.320	.03	.73	.486	.14	.489	.05	.86	.433
	Left ROI	-.25	.242				.10	.632			
Unsuccessful	Right ROI	-.07	.779	.003	.06	.940	.16	.386	0.25	5.76	.007
	Left ROI	.02	.927				.39	.036			

#### 3.4.3 Relationships between TMS-EEG measures and SSRT

For both the ADHD and control groups, the analysis revealed no correlation between SSRT and TEP (*r* = -.06, *p* = .697 and *r* = .22, *p* = .177 for the ADHD and control groups, respectively) or between SSRT and ISP (*r* = .04, *p* = .818 and *r* = -.22, *p* = .175 for the ADHD and control groups, respectively). These results indicate no direct relationship between response inhibition behavioural performance and neither the excitability of the right frontal cortex or interhemispheric connectivity.

## 4. Discussion

The aim of the current study was to investigate a functional frontal asymmetry abnormality in adult ADHD, its underlying neurophysiological mechanism and its relationship to a response inhibition deficit and clinical symptomatology. Therefore, we examined the asymmetry of the response-inhibition-related N200 ERP component and its correlation with behavioural, clinical and TMS-EEG measurements. To the best of our knowledge, this study is a first demonstration of a relationship between ERPs measured during task performance, and TEPs, measured in a non-task-related context. Furthermore, by measuring ISP, this study is one of the first to use TMS-EEG for examining functional interhemispheric connectivity in ADHD, and the first to do so outside of the motor cortex. We first replicated previous findings (mainly in children) by showing that adult ADHD individuals demonstrate diminished response inhibition performance (i.e., longer SSRT) and processing (i.e., reduced N200 amplitude) then non-clinical controls. Next, we found that the ADHD group demonstrated reduced N200 right-asymmetry, right-frontal excitability (i.e., weaker TEP), and frontal interhemispheric connectivity (i.e., stronger ISP). Finally, we showed that in the ADHD group, N200 right-asymmetry was positively related to response inhibition performance (i.e., reversely related to SSRT; for successful “Stop” trials) and to TEP magnitude (for successful “Stop” trials), and reversely related to symptoms severity (for unsuccessful “Stop” trials). However, we did not find a relationship between ISP and N200 asymmetry, while response inhibition was not found related to either TEP or ISP. As such, our results indicate that abnormal frontal asymmetry is related to a key cognitive symptom in ADHD and suggest that frontal asymmetry deficits in this disorder are underlined, at least partly, from reduced right-frontal excitability, indicated by TEP. Importantly, this result pattern favours the asymmetry hypothesis over the right-hemispheric account for ADHD, further suggesting that frontal hemispheric asymmetry deficiencies provide a more comprehensive neuromarker of ADHD and can thus better explain its symptomology than diminished right-frontal activation alone.

### 4.1 N200 and response inhibition

Across stopping success conditions, the ADHD group demonstrated diminished N200 amplitude and right-asymmetry, as well as elongated SSRT. A marginal positive relationship between increased N200 asymmetry during successful “Stop” trials and better response inhibition performance (indicated by shorter SSRT) was also observed in the ADHD, but not the control group, for which such a relationship was obtained for only right-sided N200 amplitude. However, the groups did not significantly differ with respect to the relationship between N200 asymmetry and SSRT. Taken together, these results suggest that both right-sided activation (as in controls) and hemispheric asymmetry are related to response inhibition deficits in adult ADHD. This suggests that compromised N200 frontal asymmetry is related to an inhibitory deficit specifically characteristic to this disorder. This notion is in line with previous studies supporting the brain asymmetry account of ADHD by showing increased spontaneous Alpha-band right-frontal-asymmetry in this disorder [43, 44, 46]. However, while previous studies focused on EEG measurements taken during resting-state, our results add to the accumulating evidence supporting the asymmetry account for ADHD by revealing abnormalities in brain activity specifically related to response inhibition (i.e., N200) in this disorder, and indicating a direct connection between these abnormalities and a central endophenotype of this disorder, namely response inhibition deficiency. Indeed, abnormal functional asymmetry in frontal regions has been suggested to represent an ontogenetically stable trait in ADHD [45].

Given that response inhibition is a lateralised function, which requires specialisation of the right frontal cortex [15, 21–32], it is plausible that a failure to initiate and maintain right hemispheric asymmetry may underlie poor response inhibition in ADHD, and thus be related to a core deficit in this disorder. It should be noted that our results do not rule out the possibility that frontal asymmetry may also play a role in response inhibition in non-clinical individuals, as a negative β coefficient was observed for the left frontal ROI in the control group, similarly to the ADHD group. However, it did not significantly contribute to the relationship with SSRT, suggesting that frontal asymmetry does not explain response inhibition performance beyond the mere activation of the right hemisphere in non-clinical individuals.

### 4.2 ADHD symptoms severity

As expected, symptoms severity was higher for the ADHD group than for controls. Importantly, it was found to be inversely related to N200 right-frontal-asymmetry during unsuccessful “Stop” trials in the ADHD, but not in the control group, and a significant difference between the groups was observed with respect to this relationship. This stands in some contradiction with the marginal, positive relationship found for the ADHD group between N200 asymmetry during successful stops and more efficient response inhibition (i.e., shorter SSRT). Taken together, these results suggest a dissociation between N200 asymmetry during successful and unsuccessful response inhibition in relationship to ADHD pathology: while its enhancement during successful stops is related to more efficient response inhibition processing, thus to a less severe core cognitive deficit, its enhancement during unsuccessful stops is related to more severe clinical symptomatology.

This dissociation may be explained by the added presence of error processing during unsuccessful stops. Across groups, N200 activation in the right hemisphere was stronger for unsuccessful than for successful stops. This is a typical finding in the SST [37, 77–79], which has been suggested to reflect processes related to both error monitoring and response inhibition in distinct, although partially overlapping, frontal distributed networks [77, 88–90]. Relevantly, error processing involves fronto-central symmetric activation, mainly generated by the anterior cingulate cortex [91, 92], which has been reported during unsuccessful “Stop” trials [31, 39].

Since asymmetry reflects the ratio between the activation of the two hemispheres, the addition of symmetric activation, affecting both hemispheres to a similar extant, is expected to result in reduced asymmetry. This implies that the observed relationship between stronger unsuccessful “Stop” N200 right-frontal-asymmetry and increased ADHD symptoms severity indicates that individuals showing weaker symmetrical, error processing are characterised by more severe pathology. This notion is in line with previous studies suggesting compromised error monitoring, and reduction of corresponding EEG signals (e.g., error-related negativity), in children and adults with ADHD [93–98].

The presence of error processing may also explain why no relationship was observed between right-sided activation or asymmetry of the N200 component during unsuccessful stops and SSRT for either group, as unlike for successful stops, it is not prominently reflective of response inhibition processing. Future studies should systematically examine both the unique, as well as potentially accumulating, contributions of abnormalities in both error processing and response inhibition in ADHD to the severity of its symptoms.

### 4.3 TMS-EEG

Our results demonstrate seemingly contradictive effects, with the ADHD group showing weaker right-frontal excitability but greater signal propagation. TEP reflects the summation of excitatory and inhibitory activation over stimulated neuron populations, and has been indicated to signify local cortical excitability [58, 61–64]. Here, ADHD participants exhibited weaker TEP, indicative of reduced excitability of the right frontal hemisphere, and stronger frontal right-to-left ISP, reversely indicative of functional interhemispheric connectivity [59, 65, 66].

ISP following a suprathreshold stimulation (i.e., a TMS pulse of sufficient magnitude to trigger action potentials), as measured here, has been shown to be negatively correlated with the integrity of CC fibres [47]. Importantly, these fibres constitute the main neural pathway connecting the two hemispheres [99], subserve interhemispheric inhibitory transmission, and are considered crucial for the initiation and maintenance of functional brain asymmetry [47–51]. As such, our finding of stronger ISP in the ADHD group may signify compromised frontal interhemispheric connectivity in this disorder. This notion is in line with previous TMS studies indicating aberrant interhemispheric connectivity between the motor cortices of children and adults with ADHD [100, 101], and with findings showing abnormalities in CC structure and fibres integrity in childhood ADHD, with limited evidence of them partially proceeding into adulthood [102–110].

Our results indicate that TEP, but not ISP, is related to right-frontal N200 asymmetry in ADHD adults, but not in non-clinical controls. This relationship was found to be significantly different between these groups. Relevantly, the 50–150 ms time window used here for both TEP and ISP is considered to represent the expected duration of interhemispheric inhibitory mechanisms relevant to the formation of functional asymmetry [84, 85]. Diminished excitability in a given hemisphere is expected to result in decreased inhibitory activation of contralateral circuits, and by that in reduced asymmetry [47–51]. Hence, this result pattern suggests that deficient right-frontal asymmetry in ADHD cannot be well explained by compromised CC-mediated functional interhemispheric connectivity, but by reduced recruitment of the inhibitory interhemispheric CC pathway by an abnormally unexcitable right hemisphere.

Interestingly, left-frontal negative deflection during unsuccessful stopping was found to be inversely correlated with ISP in the control group, thus positively correlated with interhemispheric connectivity. This may suggest that, when intact (i.e., in control, but not ADHD individuals), increased interhemispheric connectivity during performance of a lateralised function (i.e., response inhibition) may be related to errors; or alternatively, to increased activity related to non-lateralised functions observed during unsuccessful, but not successful stop trials (i.e., error processing; [91, 92]).

While our results did not indicate a significant relationship between functional interhemispheric connectivity and abnormal frontal asymmetry in ADHD, it should be noted that this may be influenced by the fact that the participants in our study were adults. Such a relationship may still be obtained in children with ADHD, as CC abnormalities in this disorder have been suggested to partially normalise during development [110], congruently with findings indicating that the maturation of the CC continues into early adulthood [111, 112]. Thus, future studies should similarly examine the relationships between frontal asymmetry, cortical excitability and interhemispheric connectivity in a cohort of children with ADHD. Moreover, by including structural imaging of the callosal interhemispheric pathway via methods such as Diffusion tensor imaging (DTI), future studies may shed additional light on the mechanism mediating functional asymmetry abnormalities in ADHD and its importance to the cognitive and clinical symptomatology of this disorder.

### 4.4 Clinical implications

Current ADHD treatments commonly involve chronic administration of psychotropic drugs; most commonly psychostimulants, such a methylphenidate (e.g., Concerta, Ritalin) and amphetamine (e.g., Adderall, Dexedrine). Importantly, these treatments can only provide temporary alleviation of ADHD symptoms, without producing long-term therapeutic changes in relevant brain functions. Moreover, these treatments are ineffective for about 30% of patients, have various adverse effects, are intolerable by some patients, and have a potential for abuse [113–115]. For these reasons, development of novel treatments for ADHD that can target the specific neuropathology of ADHD may be of great benefit for treating this disorder.

The results of the current study indicate a direct link between functional frontal asymmetry abnormalities and both clinical symptoms severity and a core cognitive deficiency in adult ADHD (i.e., in response inhibition), suggesting it may constitute an endophenotype of this disorder. Moreover, our results indicate reduced right-frontal excitability and aberrant frontal interhemispheric functional connectivity in ADHD adults. Thus, the development of novel treatments aimed at modulating these abnormalities may prove beneficial for the treatment of ADHD. Specifically, treatments that can normalize deficient functional asymmetry by facilitating interhemispheric connectivity, which has been indicated to subserve the transmission of interhemispheric inhibitory signalling required for the initiation and sustainability of functional asymmetry [47–51].

One example of such treatment is the use of paired associative stimulation (PAS), a TMS protocol involving pairing the stimulation of two regions through the coordination of two coils. This technique is based on Hebbian plasticity, according to which neuronal connections are strengthened or weakened depending on whether the firing of the presynaptic region precedes or follows the firing of the postsynaptic region, respectively [116, 117]. Importantly, PAS has been shown to induce plasticity in the cortex and modulation of functional connectivity, and most relevantly—lasting changes in interhemispheric functional connectivity [118–120]. Thus, PAS may prove useful for strengthening deficient frontal interhemispheric connectivity in ADHD individuals, which may in turn translate into clinical and/or cognitive improvements. Alternatively, cognitive training has been shown to increase interhemispheric functional connectivity, and may prove valuable as a potential treatment for ADHD, which would plausibly be most effective when involving cognitive tasks requiring the communication between the two frontal hemispheres [121].

### 4.5 Limitations

Late TMS evoked brain activations, like the ones used here, are known to be contaminated by somatosensory and auditory saliency artifacts which may hinder the validity of our results [122]. Thus, our ability to infer conclusions regarding the true nature of the TEP depends on the comparison between ADHD patients and healthy controls, and on the assumption that there were no differences in the auditory and somatosensory aspects of the magnetic stimulation between the two groups [123]. Indeed, the stimulation intensity used to measure TEP in the two groups was similar. It should additionally be noted that we did not use neuronavigation for TMS coil localisation. Therefore, despite our efforts to ensure consistency, we cannot rule out the possibility that small differences in the positioning and orientation of the TMS coil between different measurements influenced our results.

While the sample of ADHD adult participants in this study was comprised of individuals with various levels of medium to very high symptoms severity, as indicated by CAARS total symptoms scores, this sample was entirely comprised of university undergraduate students. Therefore, the generalizability of this study’s findings to the general population might be limited, an issue which can be addressed by future studies examining their replication in different adult ADHD populations.

### 4.6 Conclusions

To conclude, our results support the initial hypothesis regarding the existence of abnormal N200 asymmetry in ADHD and relate it to a response inhibition deficiency and clinical symptoms severity in this disorder. Thus, this study supports the asymmetry account of ADHD in suggesting that aberrant frontal functional brain asymmetry may subserve behavioural and cognitive aspects of ADHD, and therefore constitute an endophenotype of this disorder. Relevantly, our results add to the accumulating evidence indicating that frontal asymmetry abnormalities pose a more comprehensive explanation for ADHD than right-frontal deficiencies alone, and are directly related to response inhibition deficiency—a key endophenotype in this disorder. By combining TMS with EEG, we showed that this abnormality is likely underlined by reduced excitability of the right frontal hemisphere in ADHD, as well as evidence for reduced frontal interhemispheric connectivity in this condition.

Our conclusions encourage future studies to further investigate the mechanisms underlying comprised functional asymmetry in ADHD, as it is indicated here and elsewhere as central to the neuropathology of this disorder, and their importance to the cognitive and clinical symptomatology of this disorder. For example, the role of structural abnormalities in callosal interhemispheric pathways can be examined in both children and adults with ADHD by incorporating structural imaging methods such as DTI. Importantly, while the current study is focused on response inhibition, future investigations should include examination of other cognitive deficits relevant to ADHD, such as in sustained and selective attention, cognitive control, working memory, arousal, motivational processes, and processing efficiency. Moreover, our conclusions call for examination of the possibility of modulating frontal interhemispheric asymmetry as a treatment for ADHD. Future studies may explore the possibility of facilitating interhemispheric connectivity in individuals with ADHD via methods such as brain stimulation or cognitive training, and examine its relationship with alleviation in symptoms severity and/or improvement in cognitive functions related to this disorder, such as response inhibition.
